# Assessment of Knowledge, Attitudes, and Behaviors toward Eating Disorders among Adolescents in Italy

**DOI:** 10.3390/ijerph16081448

**Published:** 2019-04-24

**Authors:** Francesco Napolitano, Francesco Bencivenga, Erika Pompili, Italo Francesco Angelillo

**Affiliations:** Department of Experimental Medicine, University of Campania “Luigi Vanvitelli”, Via Luciano Armanni, 5, 80138 Naples, Italy; francesco.napolitano2@unicampania.it (F.N.); francesco.bencivenga1@gmail.com (F.B.); erikapompili@gmail.com (E.P.)

**Keywords:** adolescents, behaviors, cross-sectional survey, eating disorders, knowledge, logistic regression analysis

## Abstract

The objectives of this survey were to assess the knowledge, attitudes, and behaviors toward eating disorders among adolescents in Italy. The survey was undertaken between May and June 2017 among a random sample of 420 adolescents aged 14–20 years. Data were collected through a self-administered questionnaire. Only 22.8% correctly knew both the definition of anorexia and bulimia nervosa. Female, overweight or obese individuals, and who had at least one parent with a college degree or higher level of education were more likely to have this knowledge. More than one third (38.8%) had a fear of getting fat. Female, overweight or obese individuals, who did not know the definition of anorexia and bulimia nervosa, who avoided eating when they were hungry, and who always and usually were engaged in dieting behavior were more likely to have a fear of getting fat. Only 10.1% and 11.9% always and usually were engaged in dieting behavior, and 40.8% never did so. Respondents who usually/always had a strong desire to be thinner and who had a fear of getting fat were more likely to be engaged in dieting behavior. There is an urgent need to inform Italian adolescents about eating disorders, and healthcare workers may play a crucial role in distributing eating disorder-related knowledge.

## 1. Introduction

It is well known that eating disorders, such as anorexia nervosa, bulimia nervosa, and binge eating disorder, are an extremely complex set of conditions characterized by abnormalities in feeding patterns, extreme weight control practices, excessive concern for physical fitness, and altered perception of body image [1]. In addition, it is now an established fact that eating disorders represent one of the major public health problems due to important negative health consequences associated with comorbidities (as well as with other mental disorders) that mainly affect the cardiovascular, gastrointestinal, and genital systems [2,3]. Furthermore, suicidal behavior and mortality rates are also particularly high [4,5]. The adolescent and young adult populations are considerably vulnerable to developing eating disorders and their serious physical and psychosocial consequences [6,7]. Moreover, much epidemiological research specifically focusing on eating disorders has reported that genetic and sociocultural predispositions as well as biological and psychological vulnerabilities play an important role in their development [8,9,10].

Knowledge about eating disorders among the general population may be an important determinant to raise awareness among public opinion, healthcare providers, and institutions and to reduce the barriers to treatment and to improve the early recognition of affected cases [11,12]. Hence, a few studies have been conducted on adolescents’ knowledge, attitudes, and behaviors from different regions of the world regarding these disorders [13,14,15,16,17], but, to our knowledge, little research has been conducted in Italy concerning this topic [18,19]. Therefore, information about eating disorders could identify areas that may be targeted for future communication strategies in order to improve the healthcare services and to implement effective public health interventions and policy initiatives. 

Two objectives of this cross-sectional survey were defined. The first was to assess the knowledge, attitudes, and behaviors about eating disorders among a sample of adolescents in Italy. The second objective was to investigate the determinants influencing the knowledge level, the positive attitudes, and the appropriate behavior. A hypothesis was made that a set of individual characteristics, such as gender, age, and educational level, can contribute to the level of knowledge, that adolescents with lower knowledge are currently more likely to have a fear of getting fat, and that those who had a fear of getting fat are more likely to be engaged in dieting behavior. 

## 2. Materials and Methods

### 2.1. Setting and Study Participants

The survey was undertaken between May and June 2017 among a random sample of adolescents aged 14–20 years selected from four public high schools in the city of Naples (Italy). The sample was selected with a two stage cluster method. In the first stage, high schools were randomly selected from a list of public secondary schools in Naples. In the second stage, approximately 25% of all students were selected by simple random sampling from each school. According to a sample size calculation, 420 adolescents would be the minimum sample size required for this study to estimate an assumed prevalence of 40% of participants who had heard about eating disorders, with a confidence interval of 95%, a margin of error set as 5%, and an expected response rate of 90%.

### 2.2. Procedure

The research team first contacted the head teachers of the selected schools by email to ask them to participate and to obtain their consent. Following approval by each school, the researchers, during regularly scheduled class times, verbally explained the following to all participants: The purpose of the study, that the data collected would respect privacy and anonymity, that only the researchers would have access to the data, and that all data would be presented on a group level, so that each participant could feel free to respond because the answers could not be traced back to the participant’s identity; in addition, they were informed that they could withdraw from the survey at any time they desired for any or no given reason. The questionnaire was anonymous, and no personally identifiable information was collected. A signed informed consent was obtained from all the participants, and, for those younger than 18 years of age, the parents had to provide written informed consent. Study participants did not receive any gifts and were not monetarily compensated. 

### 2.3. Instrument

A pilot study on 20 students, which was not included in the final data analysis, was conducted to enhance clarity and simplicity, which resulted in minor modifications. The survey interview was structured to cover five question components, assessing: (1) Basic socio-demographic and general information about the students and their parents (age, gender, nationality, weight, height, number of cohabiting, educational level); (2) knowledge of eating disorders (definition of anorexia, of bulimia, and of binge eating, knowledge of the clinical complications); (3) attitudes towards eating habits (satisfaction with their weight, fear of getting fat, self-perception of their weight). Participants responded to these questions using a 5-point Likert scale from 1 to 5, with 1 being ‘‘not at all worried” and 5 being ‘‘extremely worried”. (4) Behaviors regarding eating habits. Participants responded to these questions using a 5-point Likert scale ranging from “never” to “always”. The items of the questions regarding the attitudes and behaviors of the participants were derived from the items of the Eating Attitude Test-26 [20]. (5) Eating disorder information sources. The survey itself included six questions with affirmative responses in “yes/no/I do not know” format and twelve questions with a closed and open-ended format. 

The survey questions, along with all of the study procedures, were approved by the Ethics Committee of the Teaching Hospital of the University of Campania “Luigi Vanvitelli” (protocol number 564).

### 2.4. Statistical Analysis

All data analyses were conducted using Stata statistical software version 15 (StataCorp., College Station, TX, USA) [21]. Primarily, descriptive statistics were used to summarize the socio-demographic characteristics of the sample. Secondly, multivariate logistic regression analysis was performed using the model building strategy suggested by Hosmer et al. [22]. Chi-square and Student’s *t*-test were performed for univariate analysis to evaluate the association between the outcomes of interest, and the independent variables and all factors showing a *p*-value less or equal than 0.25 were entered into multivariate logistic regression models to estimate the independent association between potential predictors and the following three outcomes of interest: (1) Knowledge of both definition of anorexia and bulimia nervosa (Model 1); (2) having a fear of getting fat (Model 2); and (3) usually/always engaging in dieting behavior (Model 3). The outcome variables were dichotomized as follows: In Model 1, respondents were divided into those who were able to correctly define both anorexia and bulimia nervosa versus all others; in Model 2, respondents were divided into those having a fear of getting fat versus all others; in Model 3, respondents were divided into those who usually/always were engaging in dieting behavior versus all others. Having a fear of getting fat and engaging in dieting behavior have been chosen as outcomes of interest since it is well known that adolescents may be mainly sensitive to their weight and the dieting behavior is common in this group and may lead to risky eating behaviors [23,24,25]. The following independent variables were included in all Models: Age (continuous), gender (male = 0; female = 1), Body Mass Index (BMI) (underweight = 1; normal weight = 2; overweight/obese = 3), highest educational level of the parents (primary school = 1; middle school = 2; high school = 3; college degree or higher degree = 4), at least one parent who is a healthcare professional (no = 0; yes = 1), having received information about eating disorders from the school (no = 0; yes = 1), and the need for additional information about eating disorders (no = 0; yes = 1). Moreover, the following variables were also included: Engaging in dieting behavior (never/sometimes/often = 0; usually/always = 1), eating diet foods (never/sometimes/often = 0; usually/always = 1), and avoiding eating when hungry (never/sometimes/often = 0; usually/always = 1) in Model 2; fear of getting fat (continuous), having a strong desire to be thinner (never/sometimes/often = 0; usually/always = 1), and feeling satisfied with their weight (continuous) in Model 3; knowledge of both definition of anorexia and bulimia nervosa (no = 0; yes = 1) in Models 2 and 3. The significance level was set at 0.2 for entering and at 0.4 for removing the variables in the stepwise logistic regression models. Odds ratios (ORs) and their 95% confidence intervals (CIs) were estimated from the logistic regression models. All statistical tests were two-tailed, and results were considered to be statistically significant if the *p*-values were less than or equal to 0.05.

## 3. Results

### 3.1. Participants

Of the 420 students randomly selected, 33 students or their parents did not provide written informed consent. A total of 387 agreed to participate and filled out the questionnaire for a response rate of 92.1%. The principal characteristics of the study participants are presented in Table 1. The sample was equally distributed with respect to gender, almost all were Italian, 17.1% had at least one parent who was a healthcare professional, two thirds were of normal weight with a mean BMI value of 21.7, and only 10.2% suffered from a chronic disease.

### 3.2. Knowledge

The correct responses regarding the level of knowledge of eating disorders of the respondents are reported in Table 2. Regarding the level of knowledge of eating disorders, 59.4% correctly knew the definition of anorexia, 39.8% correctly knew the definition of bulimia nervosa, 5.6% knew the definition of binge eating, and 7.5% were aware of the clinical complications of eating disorders. Overall, only 22.8% correctly knew both definition of anorexia and bulimia nervosa.

To identify potential independent associations between knowledge of the definition of anorexia and bulimia nervosa and several specific characteristics, multivariate logistic regression analysis was conducted and the results are reported in Table 3. The multivariate logistic regression analysis indicated that females and those who were overweight or obese were, respectively, 2.45 (95% CI: 1.44–4.17) and 3.7 (95% CI: 1.89–7.25) times more likely to know both definition of anorexia and bulimia nervosa compared with males and those of normal weight. Moreover, this knowledge—with adolescents having at least one parent with a primary school level of education as reference category—was higher among those who had at least one parent with college degree or higher level of education (OR = 2.38; 95% CI: 1.02–5.55) (Model 1).

### 3.3. Attitudes

The responses regarding eating attitudes and behaviors of the respondents are reported in Table 4. With regard to the questions on eating attitudes, 20.4% and 21.8% of adolescents were fully satisfied with their weight and how their weight influenced judgment on themselves, respectively, with an overall mean value of 3.2 and 2.9 out of a maximum score of 5. Moreover, 38.8% had a fear of getting fat—25.4% of males and 52.7% of females—with an overall mean value of 3.4 out of a maximum score of 5. The results of the multivariate logistic regression analysis showed that respondents who were female (OR = 5.79; 95% CI: 3.32–10.08), those who were overweight or obese (OR = 2.45; 95% CI: 1.18–5.09) compared with those who were of normal weight, those who did not know both definition of anorexia and bulimia nervosa (OR = 0.39; 95% CI: 0.22–0.72), those who avoided eating when they were hungry (OR = 2.62; 95% CI: 1.18–5.78), and those who usually/always were engaging in dieting behavior (OR = 2.78; 95% CI: 1.46–5.27) were more likely to have a fear of getting fat (Model 2 in Table 3).

### 3.4. Behaviors

When participants were asked a series of questions about their eating habits, only 7% reported to be always aware of the caloric content of foods, 10.9% usually avoided eating when hungry, 7.3% usually avoided eating high-carbohydrate foods, and 12.5% usually ate diet foods. Moreover, of those who usually/always were engaged in dieting behavior (21.8%), 17.3% were males and 26.8% were females. 40.8% never did so. About one fourth (27%) usually/always felt a strong desire to be thinner. The results of the multivariate logistic regression analysis showed that those who usually/always had a strong desire to be thinner (OR = 3.05; 95% CI: 1.59–5.85) and those who had a fear of getting fat (OR = 1.42; 95% CI: 1.12–1.79) were more likely to usually/always be engaged in dieting behavior (Model 3 in Table 3).

### 3.5. Sources of Information

Almost all participants (93.4%) had heard about eating disorders, and 44.9% cited parents as a good source of information, followed by friends (25.3%), mass media (21.1%), internet (15.7%), healthcare providers (8.5%), and schools (6.2%). Moreover, more than half (58.4%) stated that they needed additional information about eating disorders.

## 4. Discussion

The results presented in this comprehensive investigation offer a first insight into adolescent knowledge, attitudes, and behaviors towards eating disorders in a sample in Italy. The study was also focused on examining some possible predictors.

The results from this study highlight a lack of knowledge about eating disorders, with 59.4% and 39.8% of adolescents lacking the correct definition of anorexia and of bulimia nervosa, respectively. These observations are lower compared with the data collected in a study carried out in the United States with values of 63% and 72%, respectively [26]. One of the main reasons behind this faulty knowledge could be the incomplete and inappropriate information acquired. Therefore, identifying trusted sources for acquiring information is important because it might improve the level of knowledge about eating disorders. Though adolescents have different ways of accessing information, this study suggests that parents have been identified as the deeply trusted source of eating habits-related information. A significant association has been found between having an adequate knowledge and the level of education of the adolescent’s parents, and parents should be routinely engaged in delivering reliable and key messages. In this research, mass media and the Internet were also identified as additional sources of information. However, it is disturbing to observe that current practices of information, education, and communication of the schools did not play a primary role as an informational source for the sample, although they might be an effective channel for making decisions about correct actions. This scenario stresses the need to provide updated and accurate nutrition education in the entire curriculum in the early pre-school and kindergarten years so that the students will acquire proper information. Furthermore, it is also important to underline the facts that healthcare providers should be an important source of information and a lack of acquiring knowledge from them is indicative of a gap in communication between healthcare providers and the general population. Their educational interventions have been already acknowledged to have a pivotal role for improving knowledge and for adopting correct behaviors in different groups of the general population [27,28,29,30]. It is necessary that parents need to be acutely aware of the importance of their healthcare providers and of the need to include discussions about eating habits as a part of the routine aspect of medical encounters with their children.

A main objective of this study was to investigate the weight concerns among the surveyed sample. The results showed that only one in five adolescents was fully satisfied with their weight, and this finding is higher than that obtained in 14 to 18-year-old Taiwanese students, of whom only 12.8% were satisfied [31]. Moreover, more than one third of adolescents have a fear of getting fat, and just over half of the females have this concern. This result was similar to the finding observed in France, with 41.9% of females aged 12 to 18 years having a fear of getting fat [32]. Understanding the adolescents’ concerns with respect to body size may be useful since several studies have found that those who have an altered perception of their weight status were more likely to have inappropriate weight control behaviors [33,34,35,36].

Regarding the behavior, approximately one in five participants usually/always were engaged in dieting. Similar results were found in France and Spain [32,37]. However, it was lower than those observed in the United States, with 31% of males and 46% of females having followed diets to lose weight in the last year [38], in Iran, 39.7% of adolescent girls have attempted dieting to lose weight [39], and in Portugal, 39.8% of girls and 13.1% of boys reported dieting occasionally/often [40].

The study indicated that there are multiple factors influencing the different outcomes of interest among the surveyed population, and several aspects of the findings of the multivariate analysis merit comment. For example, in accordance with previous studies conducted elsewhere [41,42,43,44], it has been demonstrated that gender was an independent variable associated with the level of knowledge. Females had a significantly higher level of knowledge on eating disorders, and they are also more likely to have a fear of getting fat. This finding may be useful in developing specific strategies and in designing programs for improving the level of knowledge of adolescents. The variable source of information had no meaningful effect on responses. An interesting finding is that adolescents with parents who were more educated were more likely to know the definition of anorexia and bulimia nervosa. The positive impact of a higher educational level on knowledge confirmed other investigations in the same geographical area [28,30,45,46,47]. Moreover, consistent with previous studies [40,48], it has been observed that the adolescents who desired to be thinner were more likely to be engaged in dieting behavior. 

There are a number of possible limitations to this investigation that need to be addressed while interpreting the findings. First, as this study design was cross-sectional in nature, it is not possible to draw definite conclusions about causality, or even temporality, of the associations found between the main explanatory variables and the different outcomes of interest. Second, the sampling population was restricted to those from one geographic area; thus, the findings may not be generalized to the overall student population in other zones of the country. Third, because participants were asked questions about themselves, this information may have been less reliable due to reporting bias with an overestimation or underestimation of their actual attitude and practices related to eating disorders. Given that the questionnaire was self-completed and anonymity and confidentiality were emphasized during data collection, the prospect of underreporting may be limited. Despite these limitations, the findings extended previous research by identifying the knowledge, attitudes, and behaviors about eating disorders and the many factors of influence among adolescents and young adults.

## 5. Conclusions

There is an urgent necessity to inform the population of Italian adolescents regarding eating disorders. Policymakers and educators should direct their efforts firmly to enhance their knowledge, to adopt better attitudes and behaviors by providing health education and community-based interventions. Such efforts can be done through school campaigns or during general practitioner visits. Healthcare workers may play a crucial role in distributing eating disorders-related knowledge, as they are the only group involved in patient-physician interactions.

## Figures and Tables

**Table 1:** Main characteristics of the study population.

Characteristic	*N*	%
Gender		
Male	190	50.5
Female	186	49.5
Age, years	16 ± 1.6 (14–20) ^a^	
Italian Nationality		
Yes	370	96.8
No	12	3.2
Body Mass Index	21.7 ± 3.5 (14.7–37.1) ^a^	
Underweight	59	15.8
Normal weight	259	69.4
Overweight/Obese	55	14.8
Highest educational level of the parents		
Primary school	15	3.9
Middle school	44	11.4
High school	133	34.3
College degree or higher	195	50.4
Having a parent who is a healthcare professional		
No	311	82.9
Yes	66	17.1
Having a chronic disease		
No	346	89.8
Yes	39	10.2

Number for each item may not add up to the total number of the study population due to missing values. ^a^ Mean ± standard deviation (range).

**Table 2:** Responses of the participants regarding knowledge of eating disorders.

Questions	Correct Response	*N*	%
**What is anorexia nervosa?**			
Eating disorder characterized by an abnormally low body weight, an intense fear of gaining weight and a distorted perception of weight	T	230	59.4
Eating disorder characterized by eating large amounts of food with a loss of control over the eating and then purging, trying to get rid of the extra calories in an unhealthy way	F	25	6.5
Eating disorder characterized by frequently consuming unusually large amounts of food with lack of ability to stop eating	F	18	4.7
Fear of getting fat	F	13	3.3
Do not know		101	26.1
**What is bulimia nervosa?**			
Eating disorder characterized by eating large amounts of food with a loss of control over the eating and then purging, trying to get rid of the extra calories in an unhealthy way	T	154	39.8
Eating disorder characterized by an abnormally low body weight, an intense fear of gaining weight and a distorted perception of weight	F	84	21.7
Eating disorder characterized by frequently consuming unusually large amounts of food with lack of ability to stop eating	F	21	5.4
Fear of getting fat	F	10	2.6
Do not know		118	30.5
**What is binge eating?**			
Eating disorder characterized by recurrent episodes of eating large amounts of food	T	22	5.6
Fear of getting fat	F	7	1.8
Eating disorder characterized by eating large amounts of food with a loss of control over the eating and then purging, trying to get rid of the extra calories in an unhealthy way	F	5	1.4
Do not know		353	91.2

T = True; F = False.

**Table 3:** Multivariate logistic regression model indicating associations between independent variables and the outcomes of interest.

Variable	OR	SE	95% CI	*p* Value
**Model 1. Knowledge of both definition of anorexia and bulimia nervosa**				
Log likelihood = −195.21, *χ*^2^ = 28.54 (6 df), *p* = 0.0001				
Body Mass Index				
Normal weight	1 *			
Overweight/Obese	3.7	1.27	1.89–7.25	<0.001
Underweight	1.42	0.47	0.74–2.72	0.296
Female	2.45	0.66	1.44–4.17	0.001
Highest educational level of the parents				
Primary school	1 *			
College degree or higher	2.38	1.03	1.02–5.55	0.044
High school	1.69	0.76	0.69–4.1	0.246
Having received information about eating disorders from the school	1.5	0.62	0.66–3.39	0.328
**Model 2. Having a fear of getting fat**				
Log likelihood = −195.45, *χ*^2^ = 71.06 (8 df), *p* < 0.0001				
Female	5.79	1.64	3.32–10.08	<0.001
Usually/always engaging in dieting behavior	2.78	0.91	1.46–5.27	0.002
Knowledge of both definition of anorexia and bulimia nervosa	0.39	0.12	0.22–0.72	0.003
Body Mass Index				
Normal weight	1 *			
Overweight/Obese	2.45	0.91	1.18–5.09	0.016
Underweight	0.68	0.25	0.33–1.4	0.298
Avoiding eating when hungry	2.62	1.05	1.18–5.78	0.017
Having received information about eating disorders from the school	0.57	0.28	0.22–1.49	0.256
Eating diet foods	0.68	0.25	0.33–1.41	0.3
**Model 3. Engaging in dieting behavior**				
Log likelihood = −163.15, *χ*^2^ = 55.59 (6 df), *p* < 0.0001				
Having a strong desire to be thinner	3.05	1.01	1.59–5.85	0.001
Having a fear of getting fat	1.42	0.17	1.12–1.79	0.003
Knowledge of both definition of anorexia and bulimia nervosa	1.78	0.58	0.94–3.37	0.075
Feeling satisfied with weight	0.82	0.1	0.64–1.05	0.118
Body Mass Index				
Normal weight	1 *			
Underweight	0.61	0.28	0.25–1.51	0.287
Female	0.74	0.23	0.4–1.37	0.342

* Reference category.

**Table 4:** Responses of the participants regarding eating attitudes and behaviors.

**Questions**	**Response**									
	**1 (Not at All)**		**2**		**3**		**4**		**5 (Fully)**	
**Attitudes**	***N***	**%**	***N***	**%**	***N***	**%**	***N***	**%**	***N***	**%**
How much are you satisfied of your weight?	47	11.6	58	15	107	27.6	96	25.4	79	20.4
How much your weight influences the judgment on yourself?	99	25.6	82	21.3	63	16.3	58	15	84	21.8
How much have you the fear of getting fat?	80	20.7	44	11.4	53	13.7	60	15.4	150	38.8
**Questions**	**Response**									
	**Never**		**Sometimes**		**Often**		**Usually**		**Always**	
**Behaviors**	***N***	**%**	***N***	**%**	***N***	**%**	***N***	**%**	***N***	**%**
I aware of the caloric content of foods that I eat	187	48.5	96	24.9	50	12.9	26	6.7	27	7
I avoid eating when I am hungry	163	42.2	155	40.2	24	6.2	42	10.9	2	0.5
I avoid high-carbohydrate foods	202	52.5	109	28.3	38	9.9	28	7.3	8	2
I eat diet foods	137	35.5	136	35.3	44	11.5	48	12.5	20	5.2
I am engaged in dieting behavior	158	40.8	98	25.3	47	12.1	45	11.7	39	10.1
I am occupied with a strong desire to be thinner	140	36.1	83	21.4	60	15.5	24	6.3	80	20.7

Number for each item may not add up to the total number of the study population due to missing values.
